# A fuzzy system for detection of road slipperiness in Arctic snowy conditions using LiDAR

**DOI:** 10.3389/frai.2025.1600174

**Published:** 2025-07-02

**Authors:** Aqsa Rahim, Sushmit Dhar, Fuqing Yuan, Javad Barabady

**Affiliations:** ^1^Department of Technology and Safety, UiT The Arctic University of Norway, Tromsø, Norway; ^2^Department of Automation and Process Technology, UiT The Arctic University of Norway, Tromsø, Norway

**Keywords:** slipperiness detection, LiDAR, friction coefficient, fuzzy logic, Arctic region

## Abstract

The advancement of self-driving cars has significantly improved transportation by enhancing safety, efficiency, and mobility. However, their operation in Arctic environments remains challenging due to snow, ice, and slush, which negatively impact traction and road surface perception. To address these challenges, this study integrates LiDAR-based reflected intensity measurements with environmental parameters such as humidity, temperature, and the coefficient of friction to detect road surface slipperiness and roughness. A Fuzzy Logic System is developed to process these features and classify the slipperiness levels. The analysis establishes a strong correlation between LiDAR intensity and the coefficient of friction, enabling reliable detection of surface conditions. The proposed method achieves a testing accuracy of 87% in classifying road slipperiness under Arctic conditions. These findings demonstrate the effectiveness of LiDAR and sensor fusion for real-time road condition monitoring and highlight their potential in enhancing the safety and performance of autonomous vehicles in extreme weather environments.

## 1 Introduction

The Arctic region presents unique challenges for road safety and vehicle operations due to its extreme weather conditions, including persistent snowfall during winters (Padrtová, 2020). During winters, when the temperature falls below freezing, snow becomes ice, due to which roads become hazardous. Snow, rain, and fog decrease driver visibility and vehicle control (Harith et al., 2019; Bellone et al., 2021). The performance of several sensors is also affected due to low temperatures, rain, fog, high wind, and snowstorms, particularly in the Arctic region (Kramar and Määttä, 2018). The Arctic environment, with its harsh temperatures and seasonal changes, can cause some challenges for road surface detection. During the winter, heavy snow and ice might mask road features, making it impossible for standard sensors to provide accurate information (Storsäter et al., 2021; Zhang et al., 2023). When the temperature fluctuates, slush and ice might form, which causes a slippery and unsafe road scenario. These factors significantly contribute to hazardous driving conditions, making it difficult to ensure road safety (Bardal, 2017).

A major problem that is faced in the Arctic region is the slipperiness on the road; its detection is essential for maintaining safe and efficient transportation in the Arctic environment (Tennberg, 2023). Arctic roads are subjected to severe weather conditions, including freeze–thaw cycles, permafrost degradation, and heavy snow and ice accumulation, all of which contribute to surface roughness and slipperiness and compromise safe driving (Golov et al., 2022). Snow, ice, and slush create unique challenges for vehicle operation and road maintenance, significantly impacting both human-driven and autonomous vehicles (Hamid et al., 2019). In these environments, accurate and timely detection of road slipperiness and surface irregularities is crucial for preventing accidents, optimizing vehicle performance, and ensuring the longevity of road infrastructure. Traditional methods of road monitoring often struggle to adapt to the unpredictability and variability of Arctic Road conditions (Evtyukov et al., 2021). As a result, there is a growing need for more advanced detection and assessment technologies capable of providing accurate and timely information about road conditions and surface irregularities. These situations require more advanced technologies that can provide more accurate and reliable surface data.

Light detection and ranging (LiDAR) technology has been shown to be an extremely valuable approach for detecting road roughness and slipperiness, particularly in the harsh Arctic environment (Andersson and Chapman, 2011). The LiDAR intensity measures the reflectiveness rate of an item’s surface (Jutzi and Gross, 2009). These intensity levels are used to identify road elements like lane lines, signs, curbs, and pavement. Reflective materials, such as road paint, typically correspond to high intensity. Variations in intensity can be used to differentiate road surfaces from the surroundings (Guan et al., 2015). For example, the reflectance of concrete, grass, and soil is different. The mapping and maintenance of road surfaces may benefit from the intensity analysis as well. LiDAR provides a more accurate and effective method of providing information, which is extremely valuable in real-time road evaluation (Hata and Wolf, 2014). On the other hand, standard image processing algorithms are affected by weather conditions such as darkness, snow, and fog. Similarly, another important variable is the coefficient of friction, which also helps in determining surface roughness or slipperiness and can be applied to the assessment of various road conditions, such as roads covered with ice, snow, and slush (Han et al., 2016). It can measure the resistance of sliding between the automobile tire and the road surface, which is affected by minor defects (Ergun et al., 2005). A higher coefficient of friction indicates a rougher surface, which is thought to be necessary for safe driving in icy or snowy situations as it increases traction and lowers the risk of sliding (Lorenz et al., 2015). While smooth surfaces have a low coefficient of friction, they can generate reduced traction and unsafe driving conditions. Understanding the coefficient of friction is important for vehicle safety and road maintenance. Transportation institutes are improving winter driving conditions by accurately detecting and managing friction (Yu et al., 2023).

The relationship between surface roughness and the coefficient of friction is complex and can vary depending on the materials involved and the environmental conditions, such as the presence of lubrication or moisture (Guinea et al., 2024). Casassa et al. (1989) discuss the friction coefficients of snow blocks sliding on snow slopes and how they vary under different conditions. The values of the dry friction coefficients ranged from 0.57 to 0.84 and were higher than those typically used in avalanche dynamics. The friction was separated into Coulomb friction and adhesion, which is proportional to the contact area of the blocks. The author compares its findings with past research, noting that the friction coefficients obtained are consistent with previous measurements for snow blocks sliding over snow. The paper also highlights that in loose-snow avalanches, high degrees of fluidization can lead to lower friction coefficients. The friction coefficient for snow and icy surfaces was noted; it was seen that the coefficient of friction is high (in case of dry friction and sinking behavior) and low (in cases of high fluidization in loose-snow avalanches) (Casassa et al., 1989).

The aim of this study is to combine advanced sensing technology with LiDAR and coefficients of friction to measure road slipperiness produced by ice, snow, and slush in the Arctic environment. It can address some important challenges, such as evaluating the accuracy and dependability of LiDAR for detecting surface abnormalities under various Arctic weather situations to improve vehicle safety and navigation. The paper makes the following contributions:

- The study targets Arctic conditions, emphasizing the importance of detecting rapidly changing road slipperiness affected by factors such as freeze–thaw cycles, permafrost degradation, and persistent snow accumulation to enhance the safety and reliability of autonomous vehicles.
- A custom real-world dataset was collected in Tromsø, Norway, integrating LiDAR intensity, environmental parameters, and experimentally measured friction coefficients that reflect road slipperiness for the roads covered with black ice, gritted ice, fresh snow, crusted snow, and Wet Slush.
- A Fuzzy Logic-based classifier is developed, enabling explainable and adaptive slipperiness detection suitable for uncertain Arctic conditions. The proposed approach can be a valuable solution for integration in autonomous driving systems, enhancing safety in extreme environments.

The paper is organized as follows. Section 1 provides a concise literature review and motivation; Section 2 presents the materials and equipment that includes the equipment details and data collection steps. Section 3 includes the pre-processing stage, discusses the exploratory data analysis and Fuzzy Logic system that is used in this research. Section 4 includes the Results and Discussion, followed by the Conclusion in Section 5.

## 2 Materials and equipment

**Figure 1:**
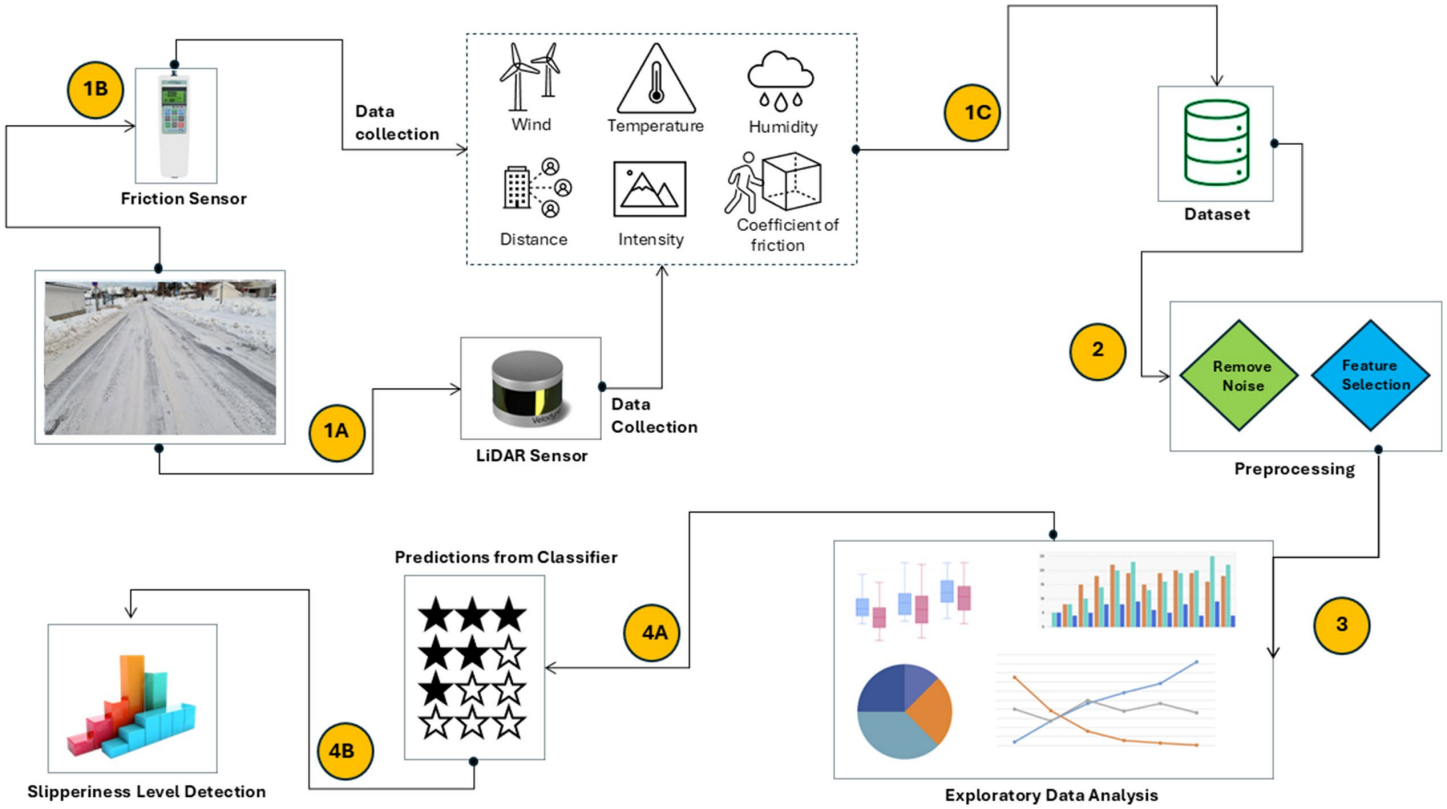
Flow of proposed slipperiness detection method.

The main steps of the proposed slipperiness detection approach are shown in Figure 1. The dataset was collected using Lidar and IoT sensors that give humidity, temperature, reflective intensity, distance and time at which data was collected. In addition to this, the coefficient of friction was also calculated by performing an experiment that used a friction sensor. After data collection, the raw data was processed to remove noise from the data and select important features to reduce the computational complexity by eliminating irrelevant features. Exploratory data analysis was conducted for the selected features to check the dependency of features on each other and with respect to the slipperiness level. Finally, Fuzzy classifier was used to predict slipperiness level on the road using the proposed features. The following sub-sections outline the entire procedure for data collection while section 3 includes the details of preprocessing, exploratory data analysis and slipperiness level detection.

**Figure 2:**
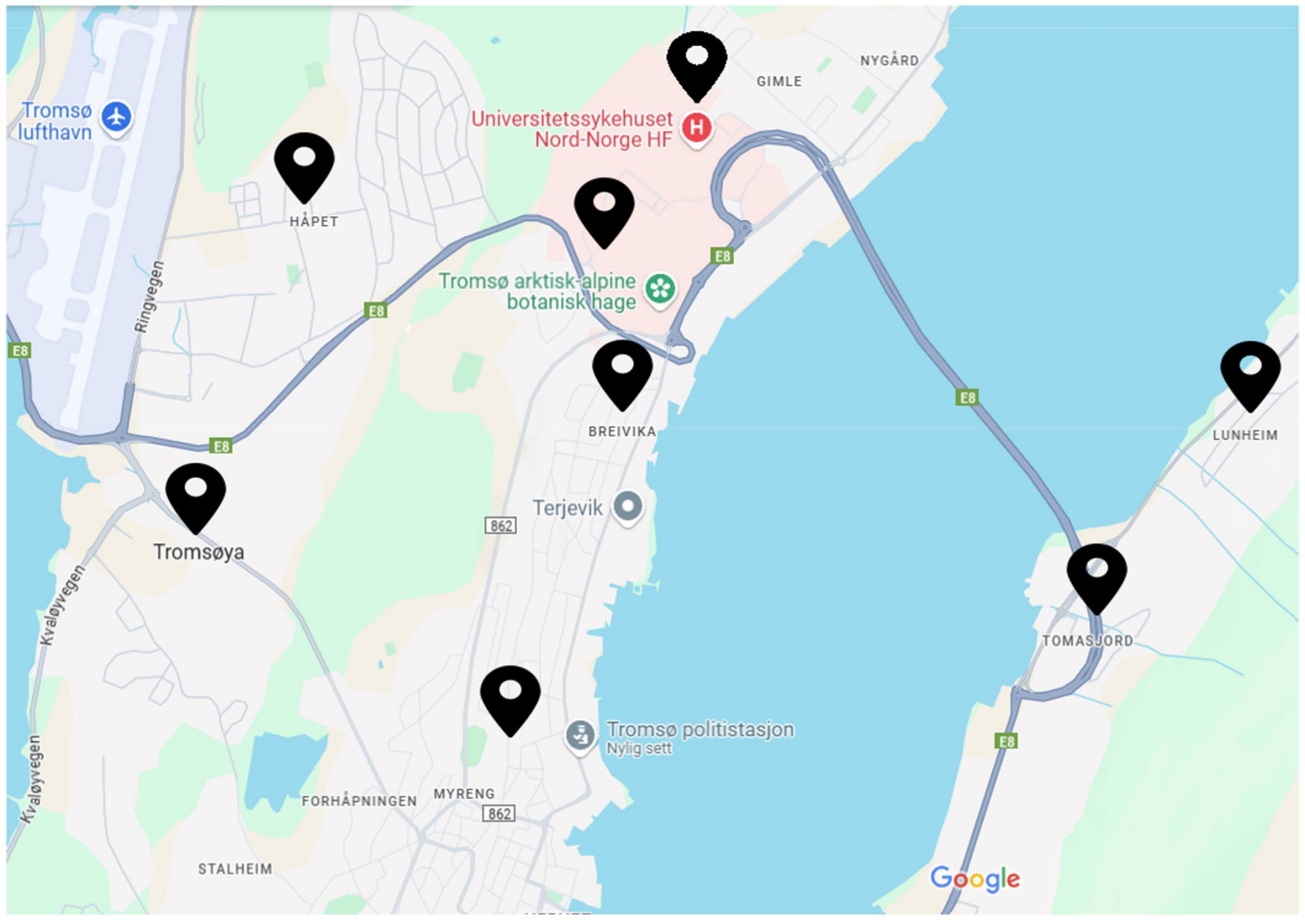
Locations for the collection of data.

**Figure 3:**
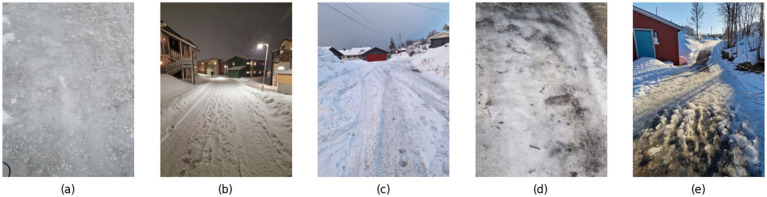
Various road conditions during data collection **(a)** road condition with black ice **(b)** road with fresh snow **(c)** road with crusted snow **(d)** road with gritted ice **(e)** road with wet slush.

An experiment was performed to collect data. The experiment was conducted for a duration of 4 months, from January to April when the temperature was ranging from −15°C to 5°C. The dataset was collected in the capital of the Arctic Circle in Tromso, Norway. The experiment was conducted at 25 different locations in Tromso, majorly including the roads in the residential area, university area, and city center, some of the main locations are shown in Figure 2. During the data collection, the roads were covered with black ice, gritted ice, fresh snow, crusted snow and Wet Slush, as shown in Figure 3. For each surface type, the data was collected for more than 1 h at different locations to include diverse samples in the dataset, as shown in Table 1. To further increase the diversity of the dataset, different kinds of roads, including normal roads that do not have any slope and roads that have some slope and zigzag nature, were considered in data collection. The dataset was collected in different weather conditions, including sunny days, rainy days and snowy days; both the day and night lighting conditions were also included while collecting the data.

**Table 1:** Details for data collection.

Surface type	Number of samples	Number of hours	Location
Black ice	19,221	4	4
Fresh snow	88,469	9	7
Crusted snow	1,732	3	3
Gritted ice	57,938	8	6
Wet slush	39,406	6	5

**Figure 4:**
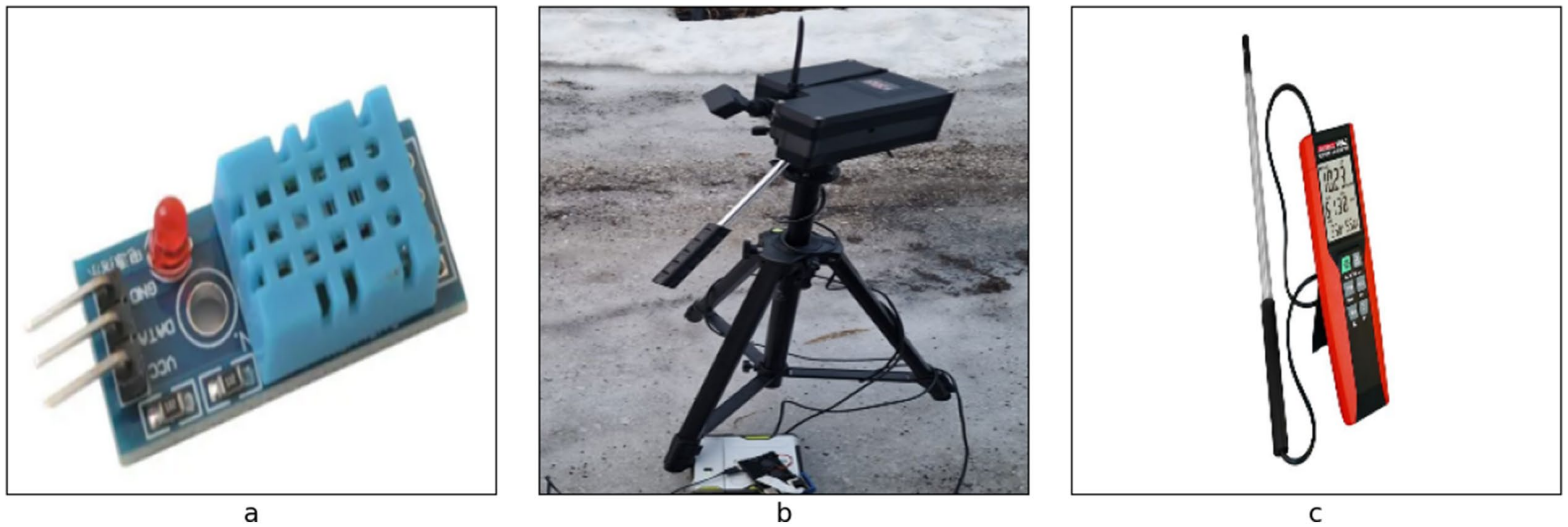
Sensors used for collection of data **(a)** DHT temperature and humidity sensor **(b)** TFmini-S12m LiDAR ranging module **(c)** hotwire anemometer.

The experiment was performed to collect the features including humidity, temperature, wind, distance, time, and reflective intensity for different surfaces including black ice, gritted ice, fresh snow, crusted snow and wet Slush. The friction coefficient is also calculated for the same surface. The values of reflective intensity, distance, and time were collected by TFmini-S12m LiDAR Ranging Module. For the temperature and humidity, DHT temperature and humidity sensor was used. Hotwire anemometer was used for collection of wind data. Figure 4 shows the sensors used for data collection. The sensors were integrated with LiDAR to give the values for the respective surface simultaneously. During the collection of the dataset, the incident angle of the sensor was also calculated. The dataset was collected at the incident angle of 84 degrees, and the distance between the sensor and the surface was normally kept at 90 cm. After every 15 min, the friction coefficient was also calculated for the respective surface to improve the credibility of the dataset. Two additional columns of friction coefficient and slipperiness level were added to the existing data file. Slipperiness level acts as a target variable and based on that, it can be decided how slippery the road is (5 being the most slippery and 1 being the least slippery). The total number of samples that were collected was 206,766. Table 2 summarizes the reflective intensity, coefficient of friction and level of slipperiness against each class.

**Table 2:** Intensity ranges across the class.

Type of surface	Reflected intensity	Friction coefficient (*μ*)	Slipperiness level
Black ice	1900–2,700	0.1–0.15	5
Fresh snow	12,600–20,000	0.21–0.22	4
Crusted snow	12,000–12,500	0.26–0.27	3
Gritted ice	3,000–6,500	0.35–0.38	2
Wet slush	7,000–10,000	0.41–0.42	1

**Figure 5:**
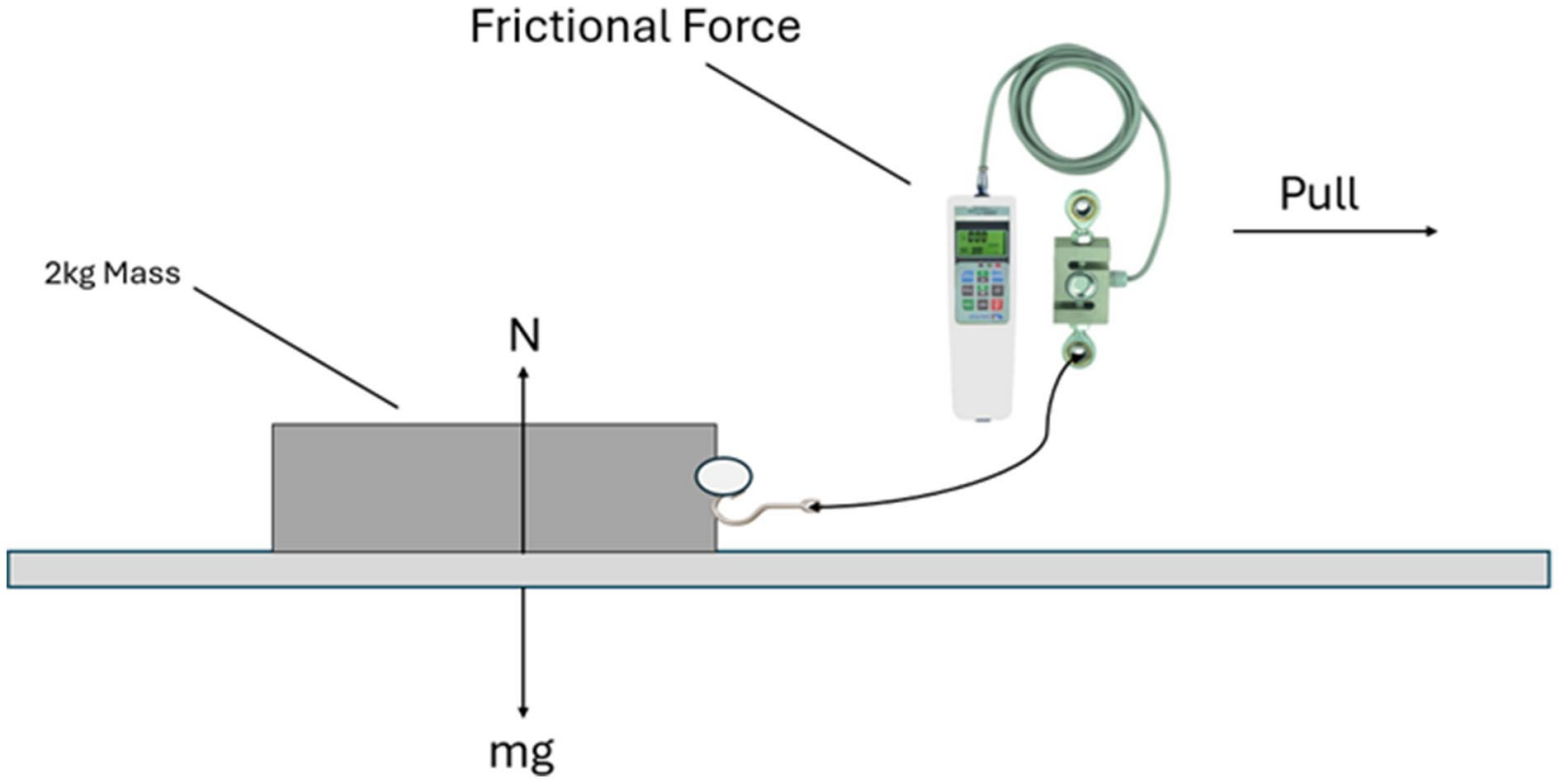
Illustration of calculation of frictional force.

The frictional force should be known to measure the coefficient of friction. To get the frictional force, the experiment utilized a 2 kg solid block with a rubber-coated base to ensure consistent material properties and simulate typical tire-road interactions, the block was placed on the road surface where the friction coefficient needed to be calculated. Attach the friction sensor to the block, while placing the block make sure it is parallel to the surface that is under consideration. The friction sensor was calibrated using a standard weight set, ensuring an error margin of ±0.05 N. A motorized pulley system applied force horizontally at a consistent speed of 0.05 m/s to maintain uniformity in the transition from static to kinetic friction. Gradual force was applied until the block began to move, with the friction sensor recording the maximum force as static friction and the stabilized lower force during motion as kinetic friction. Five trials were conducted to ensure repeatability, with average values and standard deviations calculated to validate the results. It is illustrated in Figure 5.

After getting the Frictional force, the normal force should be calculated to find the coefficient of friction (Leidich and Reiß, 2018) by using Equation 1.

(1) $$\mu =\frac{\text{FrictionalForce}}{\text{NormalForce}}\;$$

In Equation 1, the frictional force is the amount of force required to move an object from its original position, and it is parallel to the surface. While the normal force is perpendicular force exerted by a surface to support the weight of an object resting on it and is perpendicular to the surface. The normal force can be calculated using Newton’s second law stated in Equation 2.

(2) Force=massxaccleration

Where a mass of 2 kg is considered (as per experiment) and using acceleration as 9.8 m/s, the value for normal force was calculated. After getting both the values for normal force and frictional force, the values of friction coefficient (*μ*) can be calculated. All of this was for flat roads, but when the road was not flat, the slope for the road was calculated using a slope measuring application, which was used in measuring the coefficient of friction for slopy roads. Equation 3 was used in the case of sloppy roads for measuring the normal force (Pendrill, 2020).

(3) Force=mgCos(θ)

**Figure 6:**
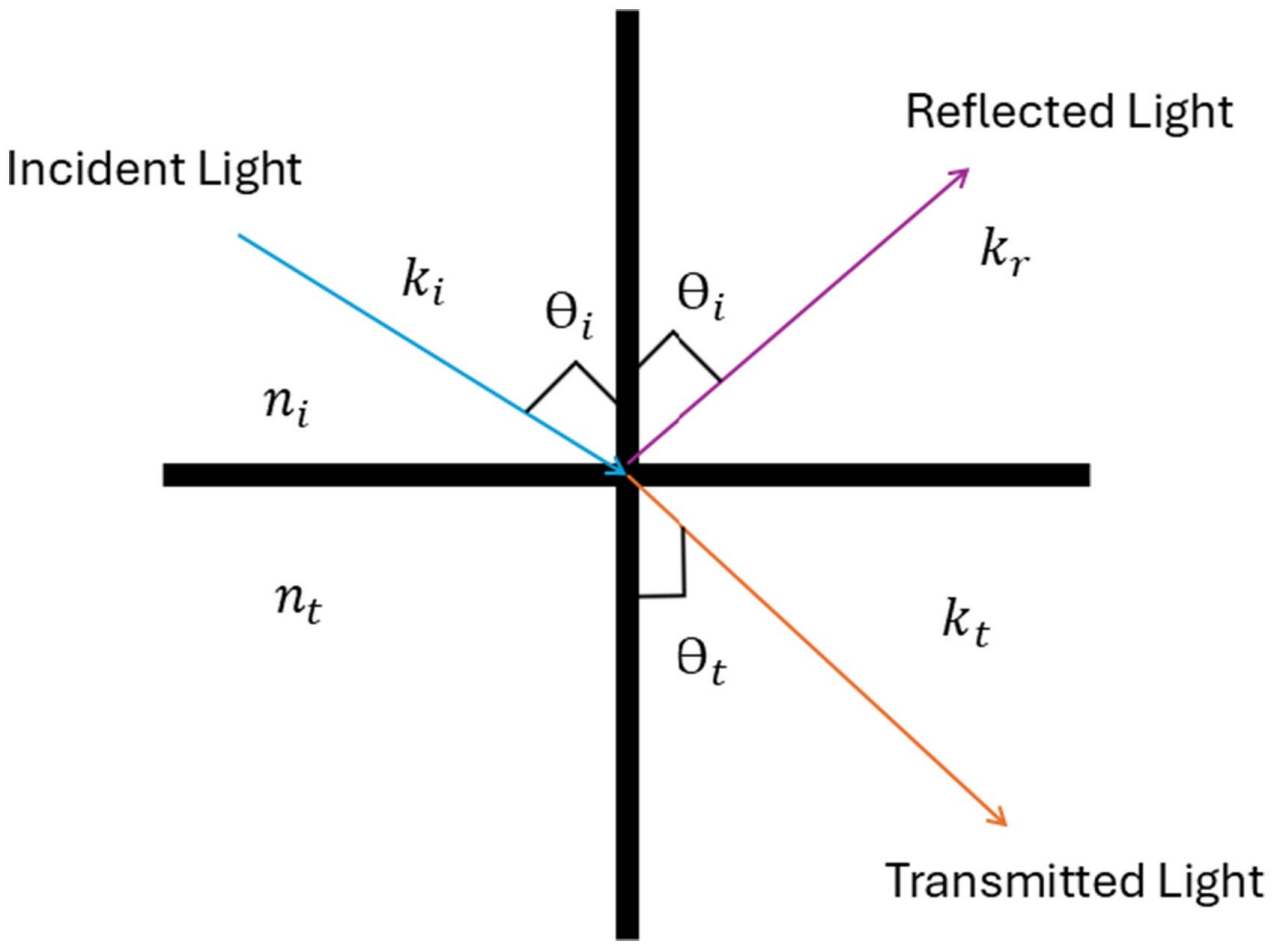
LiDAR working principle.

The other important variable is LiDAR intensity, which refers to the return reflective intensity of a laser beam in LiDAR data (Barsan et al., 2020). The value of intensities with their friction coefficient for different types of surfaces is given in Table 2. When LiDAR sensors emit laser pulses and receive their reflections, the intensity value represents the energy that is returned from the surface as shown in Figure 6. The intensity varies based on the composition of the surface reflecting the laser beam. A low-intensity value indicates low reflectivity, while a high-intensity value indicates high reflectivity. The intensity of the laser beam can also be affected by the incident angle, range, surface composition, roughness, and moisture content (Yoon et al., 2008). The LiDAR intensity can be calculated using Equation 4 (Tatoglu and Pochiraju, 2012):

(4) $$R={\frac{l_{\lambda }}{l}}_{p_{\lambda }}=z_{\mathrm{att}}(k_{d}\text{Cosθ}+k_{s})\;$$

In Equation 4, $l_{\lambda }$ is the incident intensity of the light, $l_{p_{\lambda }}$ is the intensity observed by the lidar photodetector. While z_att_ represents the attenuation of the reflection intensity due to the distance between the LiDAR and the scanned surface, and k_d_ is the diffuse reflectivity coefficient, and its value depends on the material’s properties and how it scatters light. For example, a smooth and dark surface would have a low k_d_, while a rough and light-colored surface would have a higher k_d_. k_s_ is the specular reflectivity coefficient, and its value depends on the smoothness of the surface. Smoother surfaces have a higher k_s_ value, and *θ* is the incident angle. By the Blinn-Phong Lighting Equation (Gao et al., 2019), the total intensity I at a point on the surface is the sum of the ambient, diffuse, and specular components, as mentioned in the Equation 5:

(5) $$I=I_{\text{ambient}}+I_{\text{specular}}+I_{\text{diffuse}}$$

## 3 Methodology

The collected data was in the raw form, so pre-preprocessing was done to prepare data in a clean and usable format. Feature selection was also performed to select the most important features needed to detect the slipperiness level, which helps to reduce the computational complexity by reducing the number of features as described in section 3.1. The exploratory data analysis was done to see the correlation of features with each other as described in section 3.2. The features that were selected were than used to detect the slipperiness level by using Fuzzy logic system as given in section 3.3.

### 3.1 Data pre-processing

**Figure 7:**
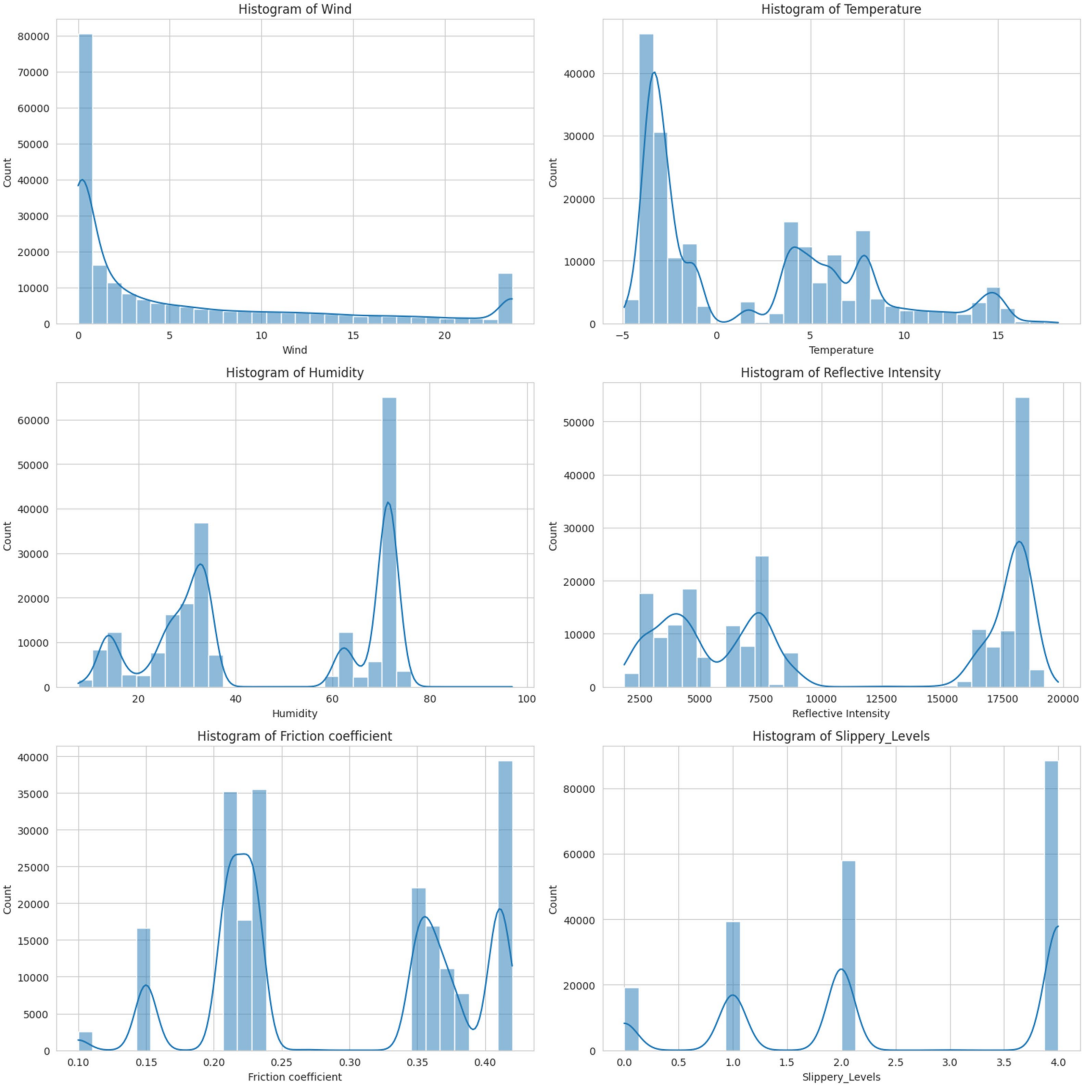
Visualization of feature distribution.

The dataset was checked for possible outliers using the histogram. The histogram for wind shows a right-skewed distribution with a long tail toward higher values. Most of the wind values are concentrated at the lower end of the scale. There were some extreme values (outliers) at the higher end. The temperature values were normally distributed, forming a bell-shaped curve. There were no significant outliers, and the values were symmetrically distributed around the mean. The humidity, reflective intensity, and friction coefficient values showed a normal distribution. The values are symmetrically distributed with no significant outliers. The slippery level showed a right-skewed distribution. Most of the values were concentrated at the lower end, with a few higher values acting as an outlier, as shown in Figure 7. The outliers were detected and removed using the interquartile range to clean the data for better understanding.

To reduce the computational complexity of the model, the irrelevant features should be removed, for that feature importance technique was used. It calculates the importance of every feature by using the decision tree and random forest. The importance of every feature is derived from how often and how effectively each feature splits the data to reduce impurity. In this case, impurity is calculated by using Gini impurity (Manokaran and Vairavel, 2023). The importance of feature j is measured as the total decrease in Gini impurity brought by that feature over all trees in the forest. Let G be the Gini impurity before the split, and G_l_ and G_R_ be the Gini impurities for the left and right child nodes after splitting on feature j, according to Equation 6:

(6) $$\mathrm{\Delta G}=G-P_{l}G_{l}-p_{R}G_{R}\;$$

**Figure 8:**
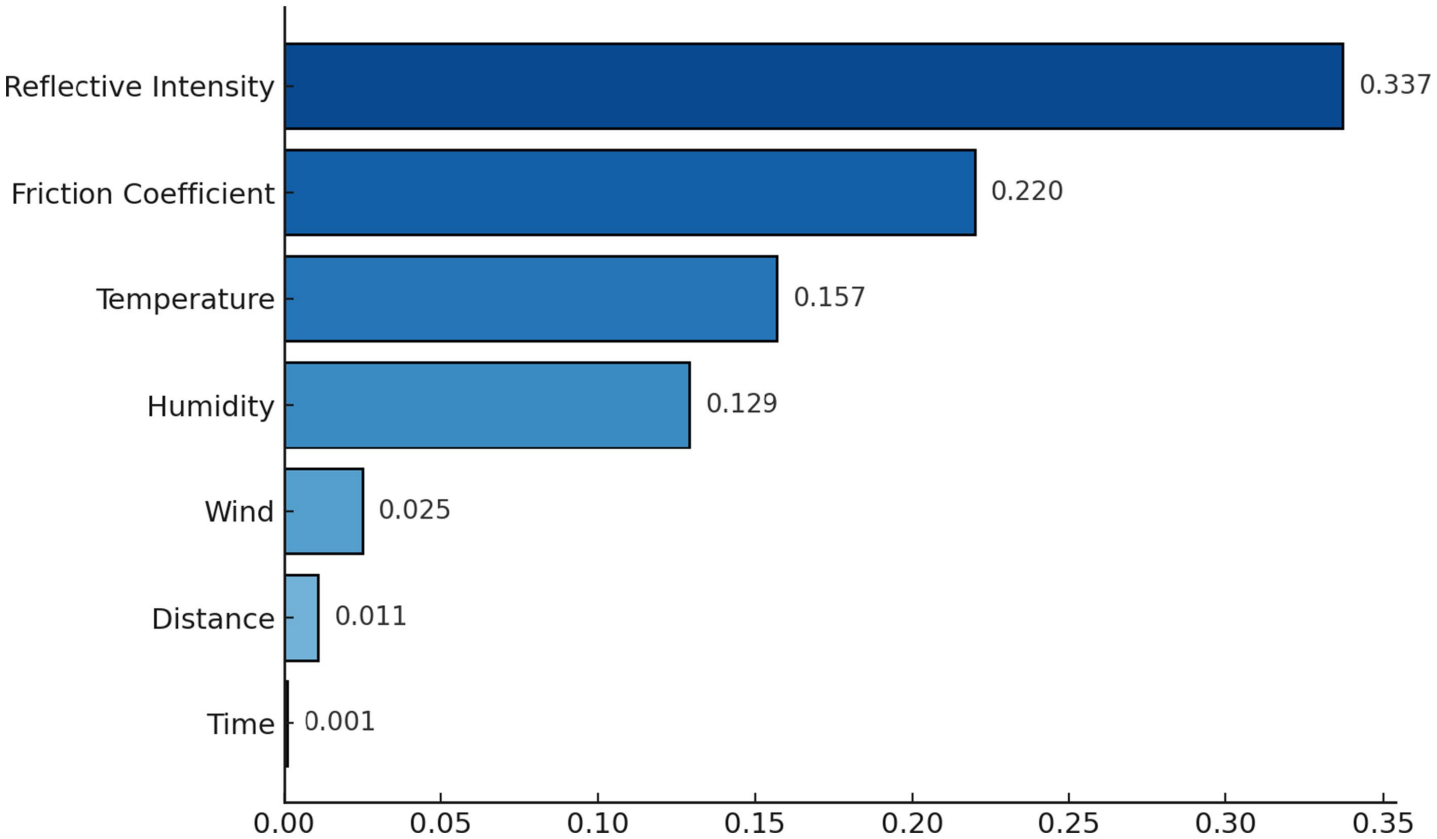
Importance of every feature.

Where p_l_ and p_R_ are the proportions of samples in the left and right child nodes. The feature importance for feature j is the sum of all ΔG values for the splits made using j across all trees. From Figure 8, the importance of features can be seen and then select the most relevant ones. When a feature is chosen for a split, the resulting reduction in impurity is recorded as the feature’s contribution. Features that reduce impurity more, especially at the higher levels of the tree, receive higher importance scores. The importance of each feature is computed as the average of its contribution across all trees in the model. The selected features were reflective intensity, friction coefficient, temperature and humidity. The fragment of the dataset by slippery level with selected features is shown in Table 3.

**Table 3:** A representative fragment of the dataset showing environmental and road surface parameters by slippery levels.

Temperature	Humidity	Reflective intensity	Friction coefficient	Slippery levels
2.2	31.3	1,927	0.1	5
2.2	31.3	1,922	0.1	5
2.2	31.3	1,927	0.1	5
−4.3	73.4	15,602	0.21	4
−4.3	73.4	15,762	0.21	4
−4.3	73.4	16,297	0.21	4
13.4	15.3	12,499	0.27	3
13.4	15.4	12,490	0.27	3
13.4	15.6	12,395	0.27	3
−1.2	32.4	4,485	0.36	2
−1.2	32.4	4,477	0.36	2
−1.2	32.4	4,486	0.36	2
8.2	29.6	7,250	0.41	1
8.2	29.6	7,256	0.41	1
8.2	29.6	7,265	0.41	1

### 3.2 Exploratory data analysis (EDA)

**Figure 9:**
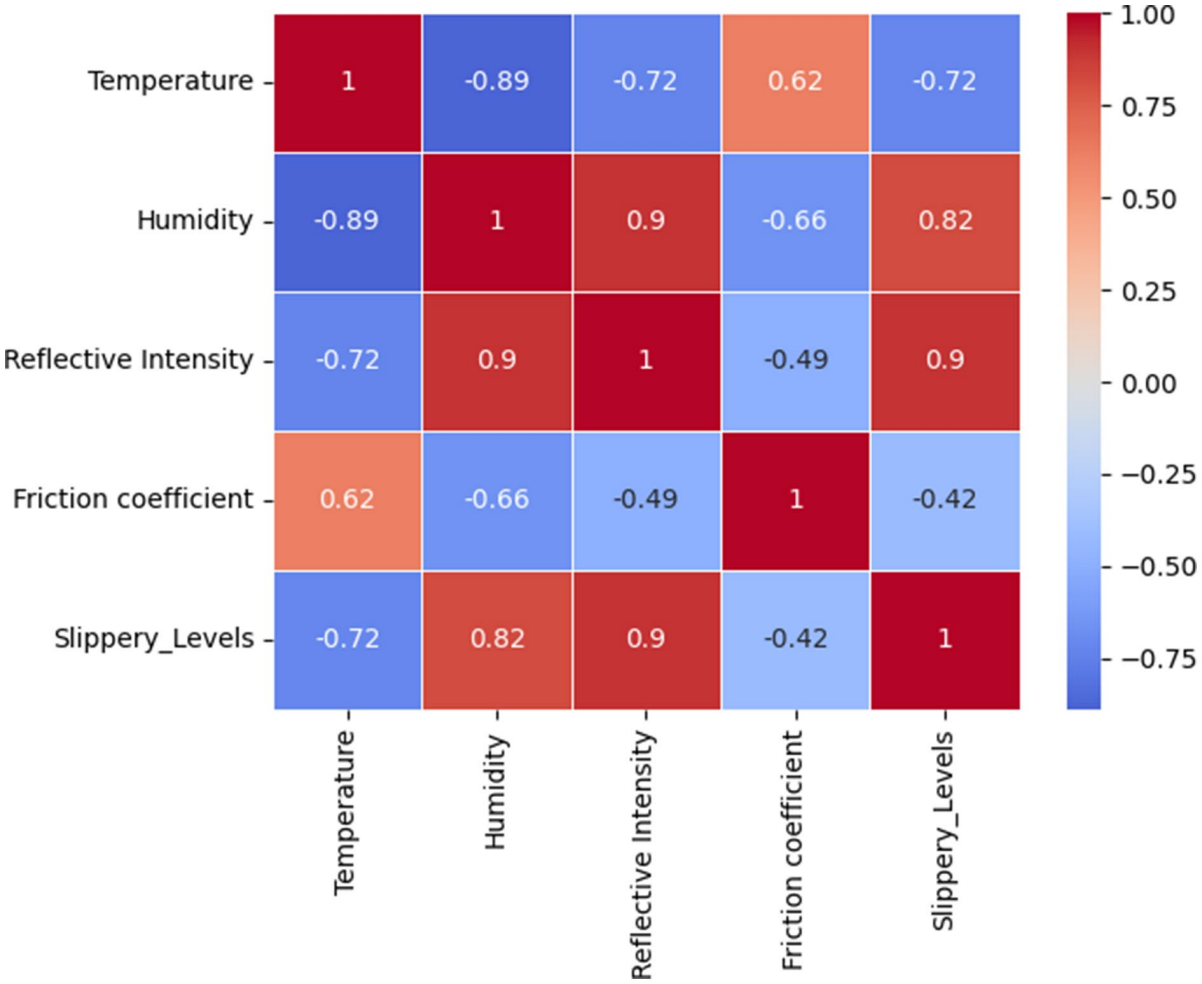
Correlation matrix.

To gain deeper insights into the relationships between collected features and their impact on road slipperiness, an exploratory data analysis (EDA) was conducted. This analysis focuses on feature distributions, correlation analysis, and statistical validation to identify key predictors for classification. A Pearson correlation matrix showed strong relationships between key variables as shown in Figure 9. Notably, reflective intensity and humidity showed a high positive correlation (r = 0.90), indicating that increased humidity enhances LiDAR reflectivity. Reflective intensity also correlated positively with slipperiness level (r = 0.82), suggesting that more reflective surfaces are typically more slippery. Conversely, temperature exhibited a strong negative correlation with reflective intensity (r = −0.72), as higher temperatures may cause surface melting, thereby reducing reflectivity. Additionally, the friction coefficient was moderately negatively correlated with slipperiness level (r = −0.42), meaning that lower friction values are associated with increased slipperiness. These findings highlight the potential of reflective intensity, humidity, temperature, and friction coefficient as key indicators in predicting road surface slipperiness.

A hypothesis was defined to verify whether each category of coefficient value has a different mean value for reflective intensity or not. H_0_ represents the null hypothesis and H_1_ is the alternative hypothesis, as defined below:

$$H_{0}=\text{Each category}\;\mathrm{has}\;a\;\text{same mean}$$

$$H_{1}=\text{Each category}\;\mathrm{has}\;a\;\text{different mean}$$

**Figure 10:**
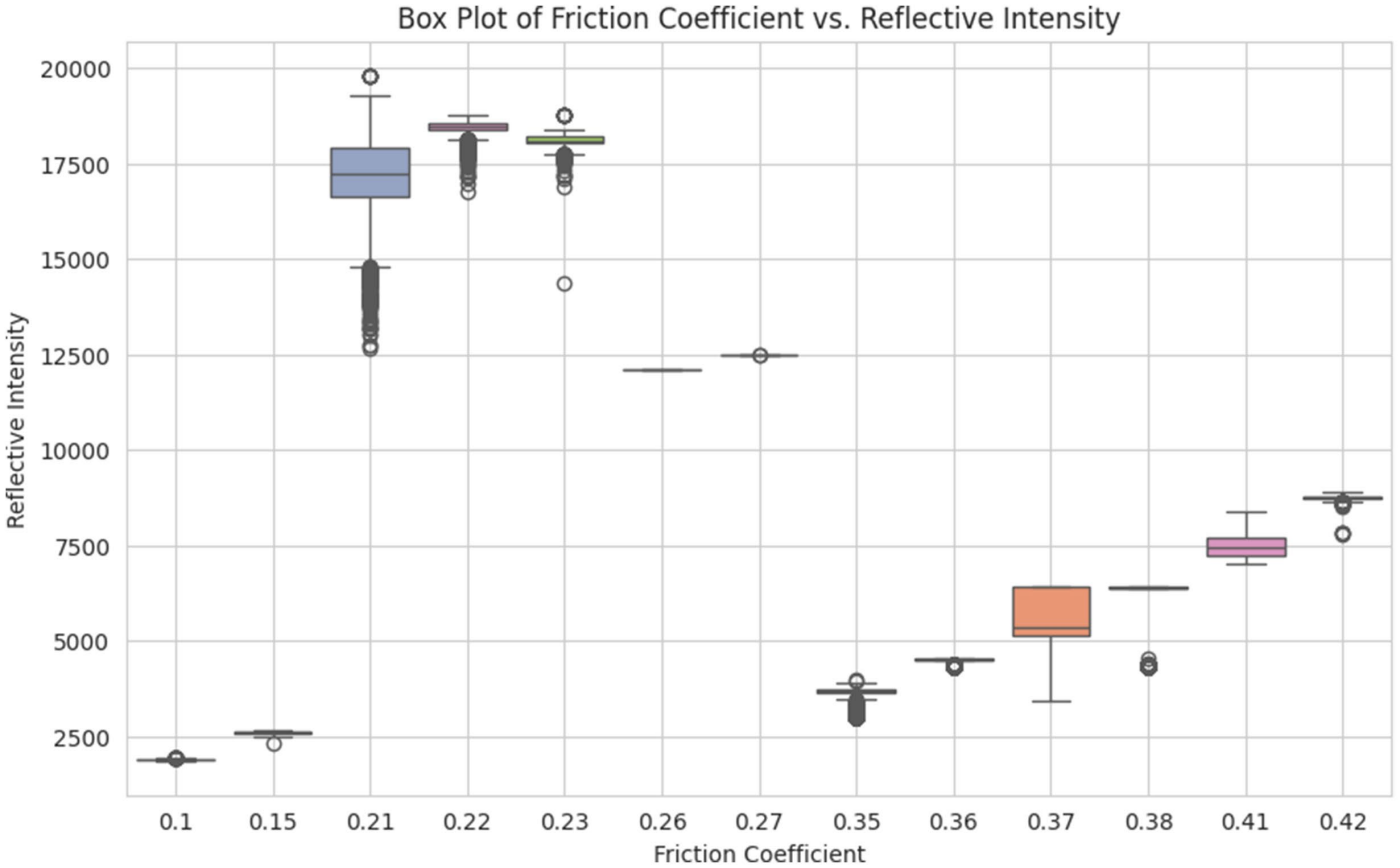
Boxplot of coefficient vs. reflective intensity.

As per the insights from the dataset, every category of slippery levels has different values of mean for reflective intensity. For example, 5’s mean lies around 2,600, 4’s mean in the range of 18,000–18,900, 3’s mean lies in 12,500, 2’s mean lies between 4,500–5,000, and 1’s mean is approximately 7,600. To statistically verify that reflective intensity varies significantly across different slipperiness levels, an Analysis of Variance (ANOVA) test (Rayat and Rayat, 2018) was performed. The results showed that slipperiness levels have significantly different mean values for reflective intensity (*p* < 0.05). *Post-hoc* Tukey’s test confirmed that each surface category has distinct reflective intensity values, supporting its role as a key predictive feature (as shown in Figure 10). Table 4 presents the results of the ANOVA test.

**Table 4:** Results of ANOVA test.

Anova test results	Sum of squares	Degrees of freedom	*F*-value	*p*-value
Slippery level	2.177639e+12	1.0	70981.79	0.0
Residuals	6.295329e+12	205201.0	–	–

The key findings are that reflective intensity and friction coefficient are the most significant predictors of road slipperiness. Humidity strongly influences LiDAR intensity, particularly in wet and slushy conditions. Temperature affects both friction and reflectivity, influencing road traction. ANOVA confirms significant differences in intensity across slipperiness levels, justifying its inclusion in classification models. These findings provide a scientific foundation for the Fuzzy Logic classifier, as discussed in the upcoming section.

### 3.3 Fuzzy system for detection of slipperiness level

The selected features, including humidity, reflective intensity, temperature, and friction coefficient, were used to detect the level of slipperiness on the road. Fuzzy logic is particularly effective in this study because it allows the system to handle varying conditions and uncertainties in a more adaptive and human-like way (Zadeh, 1975). In fuzzy sets, an element can partially belong to a set, with a membership degree ranging from 0 to 1. The membership function $\mu_{A}(x)$ defines the degree of membership of an element x in a fuzzy set A, where $\mu_{A}(x)\in [0,1]$ (Jurado et al., 2002). These functions map each input value to a corresponding degree of membership. In this case, a triangular membership function is used, which is calculated by using Equation 7, where a, b, and c define the shape and position of the triangular function (Azam et al., 2020).

(7) $$\mu_{A}(x)=\{\begin{array}{c} 0,\text{if}\;x\leq a\vee x\geq c\\ \frac{x-a}{b-a},\mathrm{ifa}\leq x\leq b\\ \frac{c-x}{c-b},\mathrm{ifb}\leq x\leq c \end{array}$$

Fuzzy rules describe the relationship between the input and output variables. Rules combine the fuzzy sets using logical operators like AND, OR, and NOT. Fuzzy inference is the process of mapping inputs to outputs using fuzzy logic rules. After that, fuzzy inputs are converted into fuzzy values using membership functions. Then, fuzzy rules are applied to compute the truth value for each rule. The results of all rules are combined to form a fuzzy output. After that the fuzzy set obtained from aggregation is converted into an output using Equation 8, where $\mu_{B}(y)$ is the membership function of the fuzzy output set (LaCasse et al., 2018).

(8) $$y=\frac{\int \mu_{B}(y).\mathrm{ydy}}{\int \mu_{B}(y)\mathrm{dy}}$$

For every feature, unique values were identified and sorted. The range for each fuzzy variable is defined by generating an array of values starting from start, up to (but not including) stop, with a specified increment (step size). This approach allows for creating a detailed and continuous range of values for each fuzzy variable, which is essential in fuzzy logic systems to accurately model the input space (Macaulay and Zhou, 2018). In the same way, the fuzzy variable for the output variable (slippery level) is defined. The ranges of input and output variables were defined according to Table 5.

**Table 5:** Range for the input and output fuzzy variables.

Fuzzy variables	Start	End	Increment
Temperature	−4.9, 18.2, 0.1	18.2	0.1
Humidity	7.6	97.0	0.1
Coefficient value	0.1	0.42	0.001
Reflective intensity	1852	19,801	1
Level of slipperiness	0	5	1

After defining the ranges, the membership function for all the features was defined to get the ranges for all variables in terms of low, medium, and high. In this case, a triangular membership function was defined. A triangular membership function is shaped like a triangle and is defined by three points: the start, peak, and end. It is essential that the entire set of possible input values for fuzzy variables are defined to make sure it handles the full scope of data (Karatayev et al., 2024). Table 6 gives the member ranges for all the features.

**Table 6:** Membership ranges for fuzzy variables.

Levels	Start	Peak	End
Temperature			
Low	−4.9	−4.9	7.5
Medium	7.5	18.2	18.2
High	20	40	60
Humidity			
Low	7.6	7.6	40
Medium	20	40	60
High	40	97	97
Reflective intensity			
Low	1852	1852	9,000
Medium	9,000	12,500	16,000
High	16,000	19,801	19,801
Coefficient of friction			
Low	0.1	0.1	0.25
Medium	0.25	0.27	0.34
High	0.34	0.42	0.42
Level of slipperiness			
Low	1	1	2
Medium	3	4	4
High	4	5	5

The correlation matrix was used to check the correlation of variables with one another, as shown in Figure 9. The correlations among the variables reveal complex relationships; temperature has a strong negative correlation with humidity (−0.89), reflective intensity (−0.72), and slipperiness levels (−0.72), but it is positively correlated with the coefficient value (0.62). Humidity exhibits a strong positive correlation with reflective intensity (0.90), and slipperiness levels (0.82), and a negative correlation with the coefficient value (−0.66) and temperature (−0.89). Reflective intensity is positively correlated with humidity (0.90), and slipperiness levels (0.90) and negatively correlated with the coefficient value (−0.49) and temperature (−0.72). The coefficient value itself shows a strong positive correlation with temperature (0.62) while being negatively correlated with humidity (−0.66), reflective intensity (−0.49), and slipperiness levels (−0.42). Lastly, slipperiness levels are strongly positively correlated with humidity (0.82) and reflective intensity (0.90), while negatively correlated with temperature (−0.72) and the coefficient value (−0.42). These correlations indicate significant interdependencies among the variables, affecting the dynamics of the system. The fuzzy rule base was constructed using four key input parameters, temperature, humidity, reflective intensity, and friction coefficient. Each parameter was divided into three fuzzy sets Low, Medium, and High based on the membership functions defined in Table 6. These inputs were used to formulate fuzzy IF-THEN rules that infer the output, i.e., the Slipperiness Level. These rules were formulated based on empirical relationships observed during data analysis and expert interpretation of Arctic Road conditions. The following rules were defined based on the correlation analysis:

$$\begin{array}{l} \text{Rule}\;1:\text{IF}\;(\text{HUMIDITY}↑)\wedge (\text{REFLECTIVE INTENSITY}↑)\\ \wedge (\text{FRICTION COEFFICIENT}↓)\Rightarrow (\text{SLIPPERYLEVEL}↑) \end{array}$$

$$\begin{array}{l} \text{Rule}\;2:\text{IF}\;(\text{TEMPERATURE}↓)\wedge (\text{REFLECTIVE INTENSITY}↑)\\ \wedge (\text{FRICTION COEFFICIENT}↓)\Rightarrow (\text{SLIPPERYLEVEL}↑) \end{array}$$

$$\begin{array}{l} \text{Rule}\;3:\text{IF}\;(\text{REFLECTIVE INTENSITY}\;\mathrm{MED})\;\\ \left(\text{FRICTION COEFFICIENT}↓)\Rightarrow (\text{SLIPPERYLEVEL}↑\right) \end{array}$$

$$\begin{array}{l} \text{Rule}\;4:\text{IF}\;(\text{TEMPERATURE}↑)\wedge (\text{HUMIDITY}↓)\wedge \\ \left(\text{REFLECTIVE INTENSITY}↓)\wedge (\text{FRICTION COEFFICIENT}↑\right)\\ \Rightarrow (\text{SLIPPERYLEVEL}↓) \end{array}$$

$$\begin{array}{l} \text{Rule}\;5:\text{IF}\;(\text{HUMIDITY}↓)\wedge (\text{FRICTION COEFFICIENT}\;\mathrm{MED})\\ \Rightarrow (\text{SLIPPERYLEVEL}↓) \end{array}$$

$$\begin{array}{l} \text{Rule}\;6:\text{IF}\;(\text{TEMPERATURE}\;\mathrm{MED})\wedge (\text{FRICTION COEFFICIENT}\;\mathrm{MED})\\ \Rightarrow (\text{SLIPPERYLEVEL}↑) \end{array}$$

$$\begin{array}{l} \text{Rule}\;7:\text{IF}(\text{TEMPERATURE}\;\mathrm{MED})\wedge (\text{REFLECTIVE INTENSITY}\;\mathrm{MED})\\ \wedge (\text{FRICTION COEFFICIENT}\;\mathrm{MED})\Rightarrow (\text{SLIPPERYLEVEL}↑) \end{array}$$

$$\begin{array}{l} \text{Rule}\;8:\text{IF}(\text{FRICTION COEFFICIENT}\;\mathrm{MED})\\ \Rightarrow (\text{SLIPPERYLEVEL}\;\mathrm{MED}) \end{array}$$

These rules are encoded in the fuzzy control system using a Mamdani-type inference mechanism. Each rule evaluates the degree of truth for its antecedents and contributes to the final fuzzy output, which is then defuzzified to obtain a crisp value for the predicted slipperiness level. The complete rule base includes rules covering all combinations of input states (Low, Medium, High) across the selected parameters. Here, only a representative subset is shown for clarity. Finally, based on these rules, a control system provides the output. A representative subset of the fuzzy rules and their structure is included in Table 7 to enhance clarity and explainability of the rule base (Phu et al., 2020).

**Table 7:** Fragment of the rule base.

Rule	Temperature	Humidity	Reflective intensity	Friction coefficient	Slippery levels
1	Any	High	High	Low	High
2	Low	Any	High	Low	High
3	Any	Any	Med	Low	High
4	High	Low	Low	High	Low
5	Any	Low	Any	Medium	Low
6	Medium	Any	Any	Medium	Low
7	Medium	Any	Medium	Medium	Medium
8	Any	Any	Any	Medium	Medium

## 4 Results and discussion

The findings from the experiment show that the friction coefficient is majorly dependent on the nature of the surface, surface roughness, and temperature. Different surfaces have different coefficients of friction. If the surface is rough, more force will be required, and it will give a higher friction coefficient. This is because rougher surfaces have more asperities or high points, which lead to greater interlocking between the two surfaces, thus requiring more force to move one over the other (Huang et al., 2024). It was also noticed that when temperature changes, it can bring changes in the properties of the material and hence, affect the friction coefficient.

In Table 2, the value for black ice and fresh snow is. 0.1–0.15 and 0.21–0.22, respectively, which is low as compared to the other types of road surfaces. It is because the surface, in the case of *μ* = 0.1–0.15, was very slippery, as shown in Figure 3a. When an object slides on black ice, there is minimal resistance between the surfaces, which results in a low coefficient of friction. Similarly, in the case of fresh snow, the surface of the road becomes smooth, as shown in Figure 3b, and less frictional force is required to move the block on that surface, which causes a low value of *μ*. Fresh snow consists of loosely packed snowflakes with irregular shapes. Although it provides more traction than black ice, it still allows relatively easy movement due to its soft and granular texture (Kabore et al., 2021). As snow ages, it compacts and becomes denser and crusted. The increased density leads to more surface roughness, resulting in higher friction. Crusted snow also undergoes partial melting and refreezing and, creating a textured surface that offers a better grip as shown in Figure 3c (Lever et al., 2019). The coefficient value for crusted snow, i.e., *μ* = 0.26–0.27, is comparatively higher because this is old snow which has added some roughness on the surface due to which frictional force that is needed to move the block on the crusted snow increases and resulted in a higher value of *μ* when compared with the value of *μ* of fresh snow. Sand or grit provides additional surface roughness, improving traction on icy surfaces, as shown in Figure 3d. The sand particles create micro-asperities that improve grip, especially for walking or driving on icy roads (Lever et al., 2019). Slush contains a mixture of water and snow particles, as shown in Figure 3e. The presence of liquid water increases the friction between the surfaces. The partially melted snow grains create a rougher surface, contributing to the higher coefficient of friction. Table 8 summarizes the friction coefficient for each type of surface.

**Table 8:** Friction coefficient for different types of surfaces.

Type of surface	*μ*	Reason
Black ice	0.1–0.15	Very smooth and Slippery
Wet slush	0.41–0.42	Wet and partially melted
Gritted ice	0.35–0.38	Roughened by sand particles, increased grip, and traction
Crusted snow	0.26–0.27	Compacted and increased compaction
Fresh snow	0.21–0.22	Smooth, light, and fluffy

Snow generally has higher reflectivity compared to black ice. This property is known as albedo (Nolin and Stroeve, 1997), which is the measure of how much light or radiation is reflected by a surface. Fresh snow can reflect up to 90% of incoming solar radiation, making it one of the most reflective natural surfaces. In contrast, black ice, especially when it is not covered by snow and has become somewhat transparent or has meltwater on its surface, tends to have a lower albedo, reflecting only about 20–40% of incoming solar radiation. The high reflectivity of snow is due to its structure, which scatters light effectively. In contrast, black ice can absorb more light, especially if it is older, contains impurities, or has a smoother surface that can form melt pools, further reducing its albedo (Flanner et al., 2021). Therefore, fresh snow has a significantly higher reflectivity than black ice, as stated in Table 1.

It was observed that by changing the incident angle, there was no change in the intensity values. The validation of the statement that sensor distance and angle of incidence do not affect intensity values was conducted through a systematic approach. A series of controlled experiments were performed. The sensor was positioned at varying distances (ranging from 10 cm to 100 cm in increments of 5 cm) and at different angles of incidence (from 0° to 90° in increments of 10°) relative to a uniform test surface with known reflectivity. Each configuration was tested multiple times to ensure repeatability. The intensity values were recorded for each configuration and analyzed statistically to identify any significant variations. A regression analysis confirmed that neither the distance nor the angle of incidence introduced measurable changes in the recorded intensity values.

For experimental verification of the proposed features, fuzzy classifier was used. The performance of the slipperiness detection model is evaluated on unseen data. The model processes unseen data by mapping continuous input variables into fuzzy sets using predefined membership functions. The system applies rule-based inference to determine the degree of slipperiness, allowing for more interpretable and human-like reasoning when handling uncertainty in input measurements. The evaluation is conducted using standard performance metrics such as accuracy, precision, recall, and F1-score by using Equations 9–12 (Khan et al., 2022) respectively, ensuring that the model can reliably differentiate between surface types as given in Table 9. K-fold cross-validation techniques was used to further assess generalization. The fuzzy classifier gave an accuracy of 87% based on the rules that were defined in Section 3.3.

(9) $$\begin{array}{l} \text{Accuracy}\\ =\frac{\text{True Positive}+\text{True Negative}}{\text{True Positive}+\text{True Negative}+\text{False Positive}+\text{False Negative}} \end{array}$$

(10) $$\text{Precision}=\frac{\text{True Positive}}{\text{True Positive}+\text{False Positive}}$$

(11) $$\text{Recall}=\frac{\text{True Positive}}{\text{True Positive}+\text{False Negative}\;}$$

(12) $$F1\;\text{Score}=2\;\frac{\text{Precision}-\text{Recall}}{\text{Precision}+\text{Recall}}$$

**Table 9:** Results for fuzzy system.

Accuracy	Precision	Recall	F1-Score
87%	85%	83%	84.5%

The proposed fuzzy logic-based slipperiness detection system offers several practical advantages for deployment in real-world Arctic driving environments. The use of LiDAR-reflected intensity in combination with temperature, humidity, and friction coefficient measurements allows the system to operate reliably under adverse weather conditions where traditional vision-based methods often fail. By providing timely and accurate assessments of slipperiness, the proposed system can be used to improve vehicle control, support early warning systems, and reduce the risk of accidents, contributing to safer and more resilient transportation networks in extreme climates. Despite the promising results presented in this study, there are some limitations of this work as well. The dataset was collected in Tromsø, Norway, which limits the generalizability of the findings to other Arctic regions with different climate patterns and road treatment practices. The technique has not been evaluated for a real-time autonomous driving application, which is an area for further work. Testing these limitations in future research will improve the effectiveness of road slipperiness detection systems in Arctic environments.

## 5 Conclusion

This study addresses the challenge of detecting road slipperiness in Arctic regions by using a Fuzzy Logic method that incorporates LiDAR-reflected intensity with temperature, humidity, and friction coefficient. It was observed that slipperiness can be effectively predicted by analyzing relationship among these features. The application of a Fuzzy Logic classifier enables robust handling of the ambiguities presented by the harsh Arctic conditions. The proposed system performs reliably incorporating different sensor inputs in extreme and uncertain environments achieving 87% accuracy in slipperiness levels classification. The main findings from this case study are presented below:

- The coefficient of friction is important for determining the slipperiness of the road and is affected by the road surface texture and weather condition. A smooth surface will tend to have lower coefficients, and a rough surface will tend to have a higher coefficient.
- The distance or angle that the sensor is positioned relative to the surface does not affect LiDAR based reflective intensity, making it a reliable indicator for road slipperiness as well as surface type.
- Reflective intensity and friction coefficient of surfaces are indirectly affected by temperature and humidity, which also contributes to the surface slipperiness.
- Each road surface type exhibits a distinct profile of friction and reflectivity, justifying their use in classification.

The proposed Fuzzy Logic model is explainable and adaptable, which allows for its real-time integration with autonomous driving systems working in the Arctic or other similarly difficult environments. Future work will target the deployment of the model with real-time autonomous driving systems and broaden the data set to cover other regions in the Arctic with varying road infrastructures and maintenance approaches. The accuracy and reliability will also be improved by studying other additional parameters and by applying sensor data fusion techniques. The proposed model paves the way toward intelligent road condition monitoring systems that can increase vehicle safety and autonomy in harsh climates.

## Data Availability

The raw data supporting the conclusions of this article will be made available by the authors, without undue reservation.
